# “We Do Exist”: The Experiences of Women Living with a Sexual Interest in Minors

**DOI:** 10.1007/s10508-021-02160-z

**Published:** 2021-11-17

**Authors:** Rebecca Lievesley, Rhia Lapworth

**Affiliations:** Department of Psychology, Nottingham Trent University, 50 Shakespeare Street, Nottingham, NG1 4FQ UK

**Keywords:** Female minor-attracted persons, Minor attraction, Pedophilia, Sexual attractions to children, Sexuality

## Abstract

The current body of the literature studying minor-attracted persons (MAPs) predominantly focuses on the experiences of men who experience sexual attractions to children. To shed more light on the experiences of women within this population, we conducted anonymous semi-structured interviews with six self-identified female MAPs, who were recruited through online support forums for individuals with sexual attractions to children. Interpretative phenomenological analysis (IPA) was used to analyze the interview transcripts. Two superordinate themes were identified from the dataset that highlighted the uniqueness of the experience of being a woman within the MAP community (“A minority within a minority”) and themes of social isolation and the effects of this on identity (“A lonely secret existence”). The findings reported here highlight how the experiences of female MAPs both converge with and diverge from their male counterparts in important ways. We discuss the implications of these experiences in relation to more effective service provision for women who are sexually attracted to children.

## Introduction

Interest in the area of sexual attractions to children or minors (referred to in this paper as “minor attraction”) is increasing in academic and social contexts. This is largely due to the theoretical link between minor attraction and sexual offending against children (Finkelhor, 1984; Seto, 2018a, 2019; Ward & Beech, 2006), but more recently has been driven by an acknowledgment that many minor-attracted persons (MAPs) live offense-free within the community (Cantor & McPhail, 2016; Dombert et al., 2016). These individuals commonly report difficulties in coping with their sexual attractions within a social context that stigmatizes them (Jahnke et al., 2015a), which leads to difficulties (e.g., perceived barriers and a lower level of willingness) in seeking professional support when this is needed (Grady et al., 2018; Levenson & Grady, 2019; Lievesley et al., 2020). That is, individuals who are sexually attracted to children often acknowledge that they would either like or need support to help them manage their sexual attractions, but fear doing so due to (1) being “outed” within society, (2) becoming the subject of community discrimination or hatred, or (3) a lack of understanding from healthcare professionals (or a combination of these things).

Although there has been a recent increase in the availability of data relating to minor attraction in the community within the literature (e.g., Dymond & Duff, 2020; Elchuk et al., 2021; Freimond, 2013; Grady et al., 2018; Houtepen et al., 2016; Levenson & Grady, 2019; Lievesley et al., 2020), this knowledge stems from samples of men within the community with such sexual attractions. In this paper we report what we believe to be the first qualitative analysis of the lived experiences of a sample of minor-attracted women.

### Defining “Minor Attraction”

The phrase “minor attraction” acts as an umbrella term to describe a range of chronophilic orientations. A chronophilia is a distinct type of sexual attraction pattern that varies as a function of the ages of preferred sexual targets (Seto, 2017). The most studied chronophilic category is pedophilia, which is defined as a primary or exclusive sexual attraction to pre-pubertal children, typically between the ages of 3 and 10 years (Blanchard et al., 2009). However, Seto’s (2017) model of chronophilias takes a much broader view and acknowledges that some people may have sexual preferences for younger infants (nepiophilia), pubescent children aged 11–14 years (hebephilia), or older minors who, depending on the legal code of a given jurisdiction, may be below the age of consent (ephebophilia). This latter category is controversial, in that some argue how some level of sexual attraction to post-pubescent minors who are approaching the age of consent is a normative form of sexuality (for a discussion, see Stephens & Seto, 2016). Seto’s (2017) chronophilias continue to encompass attractions to adults of traditional reproductive age (teleiophilia), middle age (mesophilia), and older age (gerontophilia). For the purposes of this paper, we consider “minor attraction” to encompass the nepiophilic, pedophilic, and hebephilic attraction categories.

Researchers believe that most MAPs are males (Seto, 2018a), which is consistent with work in relation to the prevalence of other forms of statistically atypical sexual interests (Joyal et al., 2015). Exploring the prevalence of minor attraction, studies have stated that 5–10% of male college students reported having sexual fantasies involving young children (Bagley et al., 1994; Templeman & Stinnett, 1991; Wurtele et al., 2014). Furthermore, a large community-based study of almost 9000 German men found that 4.1% reported having sexual fantasies involving children (Dombert et al., 2016; see also Santtila et al., 2010). Depending on the study method, these estimates of the prevalence of some degree of minor attraction can reach around 25% among men when using chat-room transcripts, where this proportion of men continued to sexualize a conversation that involved an ostensibly 14-year-old minor. That is not to say that 25% of men are primarily or even regularly sexually attracted to children, but this proportion appears to demonstrate a willingness to engage in non-contact sexual behaviors (in this case, sexualized online conversations) with individuals that they know or suspect to be below the legal age of consent. However, the more consistent prevalence estimates for minor attraction in a more clinical sense (i.e., involving directed masturbation to materials or fantasies involving children) congregate around 5% (Dombert et al., 2016; Santilla et al., 2010; Wurtele et al., 2014).

However, there is less prevalence-related research that has been conducted with women; of the few that have, it has been stated that between 1 and 4% of women declare a sexual attraction to children (Fromuth & Conn, 1997; Smiljanich & Briere, 1996). In Wurtele et al.’s (2014) work, the authors compared the prevalence of sexual attractions to children between men and women, finding that women expressed such sexual attractions at around one-quarter to one-third of the male prevalence rate (1–3% vs. 4–9%). However, a study of the prevalence of sexual fantasy use found that men and women do not statistically differ in their engagement with sexual fantasies that involve children under the age of 12 years (Joyal et al., 2015). This suggests that differences in the prevalence rates of sexual attractions to children between men and women may reflect differences in the prevalence of sexual attractions to older children or teenagers (i.e., in hebephilia). This observation is supported in the work of Bártová et al. (2021). In their wide-ranging work comparing the prevalence of paraphilias in men (*n* = 5,023) and women (*n* = 5,021) it was reported that pedophilic interest was expressed by 1.7% of men and 0.4% of women. However, when exploring hebephilic interests, the prevalence rates were 13.7% in men and 1.3% in women, reflecting a much larger sex difference. These disparities were also observed in relation to self-reported anticipated arousal to these paraphilic themes, sexual fantasy engagement, and pornography use.

### What Do We Know About MAP Experiences?

Most work with MAPs is currently confounded by conviction status, in that our knowledge of this population (specifically pedophiles, who are the usual group studied in research) is based on data from samples that are, or have been, incarcerated for sexual offenses (Capra et al., 2014; Freimond, 2013; Horn et al., 2015). Although the population of individuals with sexual convictions and the MAP community represent different groups, phrases such as “pedophile” are commonly used as synonyms for convicted populations (Feelgood & Hoyer, 2008; Harper & Hogue, 2017; Harrison et al., 2010). This conflation occurs despite research evidence showing that the number of people who experience with an attraction to children far outnumber those who have committed child sexual offenses (Theaker, 2015), and that less than half of all individuals with child sexual offense convictions meet the clinical criteria for pedophilia (Schmidt et al., 2013; Seto, 2018a). As stated previously, there is also a burgeoning evidence base into “non-offending pedophiles” (Cantor & McPhail, 2016, p. 1) and other MAPs who do not offend that suggests that many individuals live in society while experiencing sexual attractions to children (Beier, 2019; Beier et al., 2009; Elchuk et al., 2021; Jahnke et al., 2015b; Lievesley et al., 2020). Although there may be some overlap between convicted samples recruited in prisons and community samples (the degree to which this overlap exists is currently unknown due to the logistical difficulties in obtaining a “representative” MAP sample), most of the available research that uses community-based MAPs recruit from online forums that strongly condemn offending behavior. As such, although offending status cannot be completely eliminated, studying individuals with sexual attractions to children who live in the community at least minimizes the extent to which offending propensities impact the data that are collected.

Most of what we know currently comes from small-scale qualitative investigations, though these do all appear to report similar themes that indicate a degree of reliability in MAP accounts of their experiences. In one of the earliest analyses, Houtepen et al. (2016) reported how some MAPs liken their sexual development to other forms of sexuality, with an early age of recognition and a combination of both sexual and romantic attractions to children being experienced (see also Dymond & Duff, 2020; Martijn et al., 2020). According to the available literature on MAPs who are living within the community, a significant proportion of this population would like support in dealing with the psychosocial effects of living with their sexual attractions (B4U-ACT, 2011a; Elchuk et al., 2021; Lievesley et al., 2020). The most common topic of study in this regard is the experience of stigma. The effects of stigma appear to include depression, anxiety, and substance misuse conditions (e.g., Cohen et al., 2018; Elchuk et al., 2021; Raymond et al., 1999; Schaefer et al., 2010), social isolation (Elchuk et al., 2021; Jahnke et al., 2015b), and the internalization of stigma and self-loathing (Lievesley et al., 2020; McPhail & Stephens, 2020; Stevens & Wood, 2019). As an acknowledgment that these factors are known to increase the likelihood that an individual may sexually offend, some researchers have begun to discuss how MAP-directed support services should first aim to address mental health issues, with sexual offense prevention being a by-product of effective service provision (for a discussion, see Lievesley & Harper, 2021).

Exploring MAP experiences of living with their sexual attractions is a useful way of progressing social and professional discussions about the most suitable treatment approaches and targets. Common methods of coping among MAPs include the use of masturbation and sexual fantasy (Houtepen et al., 2016), internalized self-acceptance of unchosen sexual attractions (Dymond & Duff, 2020), and disclosure to others. The latter of these (i.e., disclosure) is a vital first step in obtaining external support, but many MAPs report having negative experiences when disclosing their sexual attractions (Grady et al., 2018; Levenson & Grady, 2019) and help-seeking histories are unrelated to mental health experiences (Lievesley et al., 2020). Reports of negative experiences within the MAP community lead to an inherent mistrust of professionals, which in turn results in a reluctance to seek support if or when it is needed (Dymond & Duff, 2020; Grady et al., 2018). This means that there is a need to develop mechanisms by which MAPs can feel comfortable to seek help or disclose their sexual attractions in a safe way and without judgment or persecution (Goodier & Lievesley, 2018; Grady et al., 2018; Levenson et al., 2017; Lievesley & Harper, 2021). We believe that the successful design of such mechanisms is contingent on having input from MAPs themselves, as understanding their experiences and needs should produce more responsive and effective therapeutic practices.

### The Current Study

As outlined above, there has been a relatively recent emergence of research into the MAP community, their experiences of minor attraction, and how to support them in terms of improving their wellbeing and assisting them to remain offense-free (Dymond & Duff, 2020; Elchuk et al., 2021; Grady et al., 2018; Levenson & Grady, 2019; Lievesley et al., 2020). However, this research focuses primarily on the experiences of male MAPs and therefore potentially misses important experiences of female MAPs, who represent a fringe group within this already hidden population. There is a general lack of data currently available that pertain to female MAPs. However, given that there are well-documented sex differences among adult-attracted individuals in sexual selection strategies (Buss, 1998; Buss et al., 2020; Conroy-Beam et al., 2015; Schmitt et al., 2012), preferences for short- and long-term mating opportunities (Kennair et al., 2009; Pedersen et al., 2002; Schmitt et al., 2012; Townsend & Wasserman, 2011), and sex-related emotions such as jealousy, regret, and disgust (Al-Shawarf et al., 2018; Crosby et al., 2020; Kennair et al., 2016), it makes logical sense that such differences may also exist in the experiences of people with sexual attractions to children. We also know that gay men and lesbian women differ in the extent to which their experience psychosocial adjustment issues, with lesbian women seemingly faring better than their gay male counterparts (e.g., Shenkman & Toussia-Cohen, 2020). This better adjustment (operationalized as improved self-concept and lower depression scores) may be associated with more positive social attitudes toward lesbian women than gay men (Bettinsoli et al., 2020; Herek, 2002; LaMare & Kite, 1998; Pistella et al., 2018). This may be particularly relevant to the MAP context, where negative social attitudes have been linked to stigma-related stress (Jahnke et al., 2015b), the internalization of stigma, the suppression of sexual thoughts, and reduced wellbeing (Lievesley et al., 2020), and a reluctance to seek help when it is either wanted or needed (Dymond & Duff, 2020; Grady et al., 2018; Levenson & Grady, 2019). If this reduced level of stigma toward women from sexual minorities also applies to those with sexual attractions to children, this could highlight a difference in the needs of female MAPs that is currently hidden by the androcentric nature of existing MAP research.

There are currently two published studies that report data from women who identify as MAPs, with these both presenting frequency analyses of sexual attraction patterns, the content of sexual fantasies, and female MAPs’ engagement with child abuse imagery. For example, Tozdan et al. (2020) compared a sample of 42 female MAPs to a control sample of 832 community-based women and found no differences between the groups in terms of self-reported sexual orientation (i.e., heterosexual vs. homosexual vs. bisexual), relationship status, or age. However, the MAP subsample was more likely to have engaged with child abuse imagery involving children and teenagers and had a significantly higher level of sexual fantasizing about children (with the largest between-groups difference being in relation to fantasies involving girls). Studying a smaller sample of female MAPs (*n* = 20, who were compared to 208 male MAPs), Stephens and McPhail (2021) reported how this group were less likely to be erotically attracted to girls (and more likely to be attracted to boys) than male MAPs. They were also more likely to report current adult-oriented sexual behaviors, which may correspond to a greater degree of sexual fluidity among female MAPs than men with sexual attractions to children (consistent with the broader sexuality literature that shows greater fluidity among women than men; Diamond, 2016; Geary et al., 2018; Kuyper & Vanwesenbeeck, 2009; Massey et al., 2021). However, few other differences were found in relation to sexual attractions, such as in relation to age of onset and duration of sexual attractions to children, exclusivity of these attractions, or the chronophilic orientations of their attractions. In both of these papers, a comparatively very small number of female MAPs were compared to either male MAPs or non-MAP community-based women, and the focus was on sexological features of their attractions to children. However, the experiences of living with sexual interests in children, from a phenomenological perspective, have yet to be explored.

As such, in this study we begin to address this knowledge gap by offering what we believe to be the first in-depth qualitative analysis of the experiences of women who have sexual attractions to children. It is important to note that we do not set out to compare the experiences of men and women who experience such attractions, nor do we assume similarities or differences between male and female MAP populations. In a similar vein, we do not intend to compare women with and without sexual attractions to children. Early sexological analyses of these kinds have already been presented by Stephens and McPhail (2021) and Tozdan et al. (2020), respectively. Instead, our principal aim is to offer an initial exploratory account of the lived experiences of female MAPs to inform future research studies and to provide recommendations about how to best support this group to both maximize their mental wellbeing and, where risks might exist, to prevent sexual offending.

## Method

### Participants

The participant sample compromised six adult women who had a self-identified sexual attraction to children. Participant ages ranged from late teens (inclusion criteria meant they had to be over 18) to mid-40 s. Participants were recruited internationally, with three residing in the UK and three residing in USA. Four participants were non-exclusively minor-attracted (i.e., they also reported having sexual attractions to adults), while two were exclusively attracted to prepubescent children. All participants reported that they had not engaged in any illegal behavior involving children. Countries of residence and age specific to each participant are not reported here to protect anonymity. That is, given that there are relatively few women within the already small MAP community, providing these details risks identifying individual participants within our sample. Table 1 outlines participant information.

**Table 1:** Participant information

Participant number	Exclusivity of attractions to children	Gender of attraction
P1	Non-exclusive	Female
P2	Non-exclusive	Female and male
P3	Non-exclusive	Female
P4	Non-exclusive	Female and male
P5	Exclusive	Female
P6	Exclusive	Female

### Procedure

Prior to the beginning of data collection, ethical approval was granted from the Nottingham Trent University Ethics Committee. An interview schedule was organized into three broad domains, exploring participants’ sexual attractions, methods and strategies used for managing their sexual attractions, and their experiences of disclosure and seeking support. The semi-structured nature of the interview allowed us to remain on-topic while also affording us the flexibility to explore additional matters that might arise (Smith, 2015). The aim of the interview schedule was to allow for free-flowing insights without the participant being directed by too many questions (Smith & Osborn, 2003). To maximize replicability and transparency, we have made the full interview schedule available at https://osf.io/xeshn/?view_only=013ebee24ff749b1955b0a264140b4cb.

Participants were recruited via an advert posted on Twitter and on two online forums for MAPs.^1^ Upon contacting the research team expressing an interest in the study, potential participants were provided with information sheets stating the research objectives, aims, and methods. It was explicitly specified that participation was voluntary, and participants could choose to withdraw at any point without justification. The information sheet warned of any distressing/sensitive topics that may be discussed as well as the limits of confidentiality. If participants were still willing to take part, written consent was obtained and an interview date was scheduled.

Informed consent was obtained once again at the beginning of each interview, with these conducted using either Skype audio (*n* = 5) or email (*n* = 1). Both of these methods are recognized as beneficial for interpretive researchers as they provide rich accounts of participant experiences (Curasi, 2001; Smith et al., 2009), with email interviews suggested when considering the comfort, safety, and restrictions of participants (Hawkins, 2018; Mason & Ide, 2014; Ratislavová & Ratislav, 2014). As one participant reported high levels of anxiety and distress when considering audio interview methods, email was considered to be the most appropriate option.

Each participant was interviewed once, with a mean audio interview duration of 76 min (range = 66–105 min). The audio interviews were recorded with a password-protected recording device and transcribed verbatim. The email interview followed previous guidelines and recommendations (e.g., Bowden & Galindo-Gonzalez, 2015; Meho, 2006) and involved an initial set of questions being sent to the participant via email once informed consent and rapport had been established. Once the participant had responded, additional questions were sent to the participant to follow up on previous answers, request further details or clarification on previous answers, and to cover additional areas of interest. This process was repeated until the authors had no further questions. At the end of their interview, participants received a debrief form, which provided participants with various helplines for further support. To protect confidentiality, recordings and transcripts were stored on a password-protected computer that was accessible only by the research team. Additionally, any names or places were removed from the transcripts to protect participants’ anonymity.

### Data Analysis

It is argued that interpretative phenomenological analysis (IPA) is the most effective of the qualitative methods when researching topics that are novel, emotionally laden, nuanced, and vague (Smith & Osborne, 2003; 2015), which is particularly relevant to the present study as it offers the first exploration of the experiences of female MAPs. Smith et al (2009) suggest that IPA is suited to data collection methods that allow “participants to offer a rich, detailed, first person account of their experiences” and “facilitate elicitation of stories, thoughts and feelings about the target phenomenon” (p. 56). While the most widely used method of data collection for IPA research is face-to-face semi-structured interviews, a range of other methods including audio interviews (e.g., telephone, Skype) and email interviews is considered appropriate as it is possible to generate equally rich data via these methods (Curasi, 2001; Smith et al., 2009). As such, IPA was used here to explore and make sense of the participants’ lived experiences and personal accounts (Smith & Osborn, 2003). The phenomenological focus allows understanding of conscious experiences, insights and meaning, with participants viewed as experts in the phenomenon being explored (Reid et al., 2005). IPA employs a double hermeneutic (i.e., two levels of interpretation), which involves the participants’ interpretation of their own experiences within their narratives, followed by the researcher’s interpretation of these (Smith & Osborn, 2003). It is considered that interview methods such as email may enhance this interpretation, encouraging a process of reflection throughout the interview for both the participant and researcher, and allowing the researcher to interpret the data before responding with follow-up questions (Bowden & Galindo-Gonzalez, 2015; Ratislavová & Ratislav, 2014).

The analysis was guided by previous precedents (Pietkiewicz & Smith, 2014) entailing listening to the audio recordings multiple times, verbatim transcription, multiple readings of the transcripts to ensure familiarity and noting down the authors’ primary observations of the participants’ narratives. These notes were later reviewed to establish emergent themes. The connections, similarities, and differences between the themes were then explored in order to establish clusters, and where themes were deemed to have an insufficient evidential basis, they were discarded. These clusters were then given a descriptive label—these are the superordinate and subordinate themes (Pietkiewicz & Smith, 2014). This involved an iterative process, moving between the transcripts and themes to ensure that the final themes were firmly grounded in the data and representative of participants’ accounts. A form of inter-rater reliability was also implemented—both authors analyzed sections of the transcripts independently. These were then checked by the co-author and an additional independent researcher to assess the validity of the interpretations being made (Willig, 2008).

## Results and Discussion

This paper unpacks two superordinate themes and the associated subthemes that were identified through the process of analysis. Table 2 provides an overview of these themes.

**Table 2:** Overview of themes

Superordinate themes	Subordinate themes
1. “A minority within a minority”	1.1. Questioning existence 1.2. Double-edged sword
2. A lonely secret existence	2.1. Hidden part of identity 2.2. Alienation and loneliness 2.3. Disclosure deterrent

## “A Minority Within a Minority”

### Questioning Existence

All participants discussed how many people do not recognize the existence of female MAPs, suggesting that they are hidden in social discussions about minor attraction:

Honestly I think it's more expected from guys. Yeah I mean I mean most paedophiles are just depicted as being men. Yeah. When it comes to women I think there’s a lot of people that say you know female pedophiles don’t exist.

(Participant 4)

I don’t think people are aware of the fact that females can be pedophiles too but we do exist…Women are not known to be pedophiles. If a woman is a pedophile, it’s rare for her to say so. The stigma is that “all pedophiles are men”

(Participant 3)

This observation of a lack of awareness about women within the MAP community is reflective of both the existing literature on minor attraction on the one hand and social stereotypes about sexuality on the other. To our knowledge the work presented in this paper is the first formal examination of the lived experiences of women with sexual attractions to children. Previous work has either only included men as participants (Cacciatori, 2017; Dymond & Duff, 2020; Freimond, 2013; Houtepen et al., 2016), has involved larger quantitative analyses of surveys wherein women make up only a minority of each sample (Cohen et al., 2018; Elchuk et al., 2021; Lievesley et al., 2020; McPhail & Stephens, 2020), or has only studied descriptive sexological features of sexual attractions to children among female MAPs (Stephens & McPhail, 2021; Tozdan et al., 2020). This lack of focus on minor attraction across the gender spectrum seemingly risks alienating women within the MAP community, while simultaneously limiting our understanding of this population at a broader level (Goode, 2010).

There is an established literature that suggests women demonstrate higher levels of sexual fluidity than men (for a review, see Diamond, 2016). It may therefore be that more minor-attracted men are exclusively (or predominantly) attracted to children (and thus not to adults), with recent research finding the rate of exclusive minor attraction (operationalized as the endorsement of chronophilias below teleiophilia) is somewhere between 50 and 75% (Elchuk et al., 2021; Lievesley et al., 2020). In contrast, minor-attracted women might experience such attractions more incidentally (or as part of a broader attraction pattern that also encompasses adult targets). Other analyses have reported that female MAPs are more likely to be engaged in sexual behaviors with adults in comparison with male MAPs (Stephens & McPhail, 2021). Engaging in adult-adult sexual relationships may help female MAPs remain hidden within the community and appear teleiophilic (i.e., attracted to adults) to their friends and families. The increased rates of adult-directed sexual activity among female MAPs might also explain the apparent higher rates of minor attraction among men than women among community MAP samples, with the former viewing this as a dominant theme of their sexual identity and, subsequently, being more likely to seek support in online communities. This could indicate that there is potentially a higher rate of sexual attractions to children among women within the general community than currently known, with such individuals being able to hide this (or perhaps even not recognizing their minor attraction) due to their maintenance of sexual relationships with adult partners.

In addition, media representations of MAPs often evoke the stereotype of the “predatory male pedophile” (King & Roberts, 2017, p. 72), further concretizing the view that the MAP community is predominantly or exclusively made up of men. For some of our participants, this denial or uncertainty surrounding the existence of female MAPs had an impact on their own beliefs:

…we can't really say how what proportion of paedophiles are female. It's like I don't think there's ever, well there has not been a study on that and you can't really determine that from the forum but erm but even even in regards to that there have been times when I doubted my my own mind in that way because because I had been just doing so much research and had pretty much come across nothing on paedophilia in women. I doubted in my own mind if pedophilia exists in women and I had even came across researchers saying that it might not exist in women, which made me wonder for a bit of time whether it was all in my head.

(Participant 5)

This extract highlights the doubt and confusion instilled in participants regarding their sexual attractions as a result of a lack of acknowledgment of female MAPs more broadly. This appears to have had a particularly profound effect on Participant 5. The doubt surrounding the legitimacy (or realism) of her sexual attractions to children, coupled with her lack of attraction to adults, caused emotional turbulence and the feeling that her issues may be “all in my head.” Joining an online forum and discovering other female MAPs were therefore an important point for all participants in confirming and validating their existence:

I had got to such a desperate position. I kind of didn't care anymore or I went on the Internet thought fuck it. If I die I die. But that was at the same time when I started thinking about therapy and everything. But that is after 26 years of keeping it secret…But yeh it helps seeing that there’s others. It really helps seeing that there is other women there and there is loads which I didn’t expect, I thought I was the only one so it’s a bit of reassurance that you’re not alone.

(Participant 1)

Whenever there’s a new female member on the online forum, one of the first things they say is “wow there are other women here, I didn’t expect that. I thought I was pretty much the only one in the world”.

(Participant 5)

These extracts accentuate the self-doubt and confusion they felt at times as a result of the internalized belief that female MAPs do not exist. The discovery of others was therefore met with surprise, but ultimately provided a sense of comfort and confirmation that what they had been feeling or experiencing was both real and shared by others. The extracts presented here echo the narratives and experiences of male MAPs in previous work (Dymond & Duff, 2020; Elchuk et al., 2021; Houtepen et al., 2016) and are further explored in Theme 2 (below) and emphasize the isolation and loneliness that our participants had at times felt. However, the recognition that they were “not alone” allowed the participants to overcome some of these emotions, easing the loneliness and providing reassurance and support.

Being friends with her online has helped enormously because she is really really clever and you can tell she is really intelligent… I have got her online and she is beautiful and she is successful and is clever and it made me think, wow ok people like us aren’t just hideous monsters and it has been extremely helpful and helped me keep positive for my future.

(Participant 1)

Here, Participant 1 discusses the positive impact of developing a friendship with another female MAP online. Her use of “like us” suggests that all women with a sexual attraction to minors are the same, and that their attraction is part of what defines them. It is clear that she had previously had a very negative perception of herself and others who share her attraction patterns, and while viewing others in a positive light has allowed her to reconsider and challenge this view, she has not completely moved past it with the recognition that they “aren’t just hideous monsters.” This internalization of social stigma is not a new phenomenon, having been observed in a range of marginalized sexuality groups, and recently among MAPs. These internalized feelings of abnormality and self-discomfort often lead to negative mental health outcomes, including depression, anxiety, substance misuse disorders, and suicidality (Austin et al., 2017; Heiden-Rootes et al., 2020; Szymanski et al., 2008). Among MAPs, McPhail and Stephens (2020) have developed an internalized pedo-negativity measure that purports to quantify the internalization of social stigma among the minor-attracted community. They have found that higher scores on this measure are predictive of poor psychological wellbeing and substance misuse issues (see also Elchuk et al., 2021; Lievesley et al., 2020). For Participant 1 in particular, building a positive relationship with someone she views as being successful while being minor-attracted allowed her to see that it is possible to incorporate her sexual attractions to children into a prosocial and successful broader identity, and this helped her to feel some positivity toward her future. The forensic literature has made a recent move toward seeing people as more than just what they have done, or what their sexual attractions are (Seto, 2018b; Willis, 2018). However, the narratives of the women in our sample suggest that having positive role models that show how a full life can be lived with minor attraction instills hope, which in turn is associated with greater levels of wellbeing among MAPs (Lievesley et al., 2020). This observation is consistent with the narratives of male MAPs in previous work, who suggest that gaining support from fellow MAPs is an effective way of managing one’s own negative emotions, coping with one’s sexual attractions, and reducing sexual risk (Freimond, 2013; Houtepen et al., 2016). Although this consistency across the literature is encouraging, treating female MAPs in the same way as men within the MAP community risks ignoring some of the unique challenges facing this group.

### A Double-Edged Sword

The participants in our sample discussed both positive and negative aspects of being a woman with sexual attractions to children. While the experience of being a member of the MAP community is one that is often fraught with feelings of difference from the rest of society (Jahnke et al., 2015b), the female MAPs in our sample discussed feeling like they are further isolated and excluded because of their gender. Throughout the narratives, the majority of participants discussed how it felt being part of a population that is dominated by males:

I joined an online forum years ago… I was the only female on there and I was kind of always seen as the odd one out and there is definitely a feeling of being a minority within a minority and er…I have always felt kind of you know, like I am a double freak because of it…there was one or two users who doubted I was a woman…like a lot of them are a little bit suspicious if you mention that you’re a female and they don’t necessarily believe we exist, so that’s something that has been frustrating and alienating.

(Participant 5)

There are quite a lot of women on the forums now but hardly any in comparison to men…we’re definitely outnumbered which is sometimes hard because they don’t always understand it from our perspective, or understand what it’s like to feel ignored or not have the same level of support or connection to others because there’s so many of them.

(Participant 6)

The extracts here echo the sentiments emerging from previous work with MAPs that identify loneliness, isolation, and internalized stigma as key parts of the MAP experience (Dymond & Duff, 2020; Elchuk et al., 2021; Freimond, 2013; Houtepen et al., 2016; Lievesley et al., 2020). However, the points raised here identify a certain level of stigmatization of female MAPs from within their own community, exacerbating the feelings of loneliness that they might experience throughout the course of their life. Participant 5’s labeling of herself as a “double freak” is particularly telling, adding layers of intersectional stigma to her identity.

In addition, for some participants they were also marginalized due to the gendered nature of their sexual attractions:

I have noticed that the majority of women online are mainly attracted to young girls…So at times I can feel like an alien as my attractions are 80% younger boys, 15% girls and 5% adults, so when I am in a thread where no one is attracted to young boys I feel a bit of an outcast. So at times I can almost feel more alienated there than if it was all men who were attracted to younger boys, if that makes sense.

(Participant 2)

Here, Participant 2 discusses feeling different to other female MAPs for not sharing the same attractions to girls. Her observation about the targets of sexual attractions among female MAPs is not necessarily supported by the limited evidence currently available (according to Stephens and McPhail (2021), women in this population appear to be more likely to be attracted to boys than girls, with the same rate of opposite-sex attraction also observed in male MAP groups), but is consistent with the genders of attraction seen in our sample. By referring to herself as “an alien” she is suggesting a perception of stark contrast between herself and others, and the feeling that she does not belong. While this has more of an internal emphasis, she then goes on to describe herself as “an outcast.” This experience of perceived social exclusion, alienation, and discrimination is commonly reported by other sexual minority groups and has been identified as a key factor driving mental health disparities, substance use disorders, and suicidality (Gevonden et al., 2014; Grella et al., 2009; Meyer et al., 2008; Warner et al., 2004). What is of concern is the potential for internalizing these feelings. That is, if individuals are seeking support for their sexual attractions and are then rejected or marginalized by the community that is supposed to understand, this leads to an increased risk of further mental health difficulties related to confusion about one’s identity and ties to others. When combined with the experiences of Participant 5 above, this potentially has significant implications for the design of safe spaces for female MAPs to find support. That is, these narratives might suggest that there is a need to develop specific forums for women with sexual attractions to children to seek and offer support with others.

Some participants discussed the positive aspects of being a woman with this attraction, with the view that it was in some way considered “a softer blow” (Participant 5) than for males with the same attraction.

I would say that the stigma is higher for men and I think this is because people believe that men are more aggressive or have a higher sex drive. But at the same time I don’t think I have really heard about like female pedophiles in the media or anything, it’s all males…I don’t think there can be a stigma towards something you don’t know exists, but if they did know then the hatred would be just as bad.

(Participant 2)

I think there’s actually less stigma attached towards females and it is not quite as bad for some reason or they don’t realize that they exist.

(Participant 4)

The extracts here highlight how participants felt that misconceptions, and a lack of understanding from society, contributed to less stigma toward them in comparison with male MAPs, with the view that “it is not quite as bad.” Participant 2 attributes this to public perceptions of increased aggression and sex drive in men, in comparison with women, again skewed by societal stereotypes and media representations of sexual crime (Harper & Hogue, 2017; King & Roberts, 2017). However, this view of a more lenient approach to women is then discarded when considered in light of the possibility that others simply do not know they exist (as discussed in Theme 2), but should they know then the response would be comparable. Indeed, some individuals who have less rigid views about sexual crime may actually express more punitive attitudes toward women with sexual attractions to children. In one study, Harper and Bartels (2018) reported how participants with incremental (or malleable) beliefs about sexual crime recommended more severe punishments for female perpetrators of child abuse than male perpetrators. The exact reasons for this were not explored, but one potential explanation is tied to the perception of “double deviance” (Harper & Bartels, 2018, p. 289), linking to the earlier “double freak” attribution made by Participant 5. Bringing these findings together it appears that societal perceptions about the role of gender in sexuality exacerbate the internalization of stigma among female MAPs and provide additional layers of difficulty in understanding and coping with their sexual attractions.

## A Lonely Secret Existence

### Hidden Part of Identity

Within their narratives, participants expressed how they felt that they were being secretive by not sharing what they thought to be a core aspect of their identity with others that they cared about. Despite this, some felt unable to reveal their sexual attraction.

It does feel a little bit like I have to hide a part of myself from people and I’m usually a very very open person, so I found that a bit odd for me to hide something that I consider to be important that I’m not telling anybody and it sometimes feels that I am living a lie.

(Participant 2)

The feeling of living deceitfully, because in a way it is a terrible deceit to be with someone you love so much and not tell them your deepest darkest secret is a worry. It is a deceit and that feels horrible, but at the same time I want a partner and I want to be relatively happy as I can be in life.

(Participant 1)

The extracts above highlight how both participants felt that they had to hide a part of their identity, part of themselves, by withholding their sexual attraction to maintain relationships with others. This is similar to the experiences of individuals who have other marginalized sexual identities, including those who comprise the LGBT spectrum. That is, many individuals within sexually marginalized groups may seek to conceal their identities to try to avoid experiencing discrimination or rejection (Pachankis et al., 2020). Although this concealment may be seen as an initial coping strategy among these groups, hiding one’s sexual identity is correlated with adverse mental health outcomes such as anxiety, depression, and substance misuse issues (Pachankis et al., 2020; Ragins et al., 2007). Of course, the experience of minor attraction (as a sexual attraction pattern directed toward a population who both legally and morally cannot consent to sexual activity) is different to other sexual minorities within which consensual sexual activity is both legal and a healthy route to sexual satisfaction. In drawing such a comparison we do not draw any parallels about the legality or morality of acting on any form of sexual attraction, nor do we suggest or advocate for any correlations between LGBT identities and sexual attractions to children. What we do refer to, though, are the similarities within the lived experiences of individuals who live with sexual attractions that are subject to stigmatization.

For our participants, the effects of the concealment of their sexual attractions on how they view their peer relationships may be linked to gendered norms related to social styles. That is, men typically build larger and more socially diffuse networks, whereas women (on average) tend to prefer smaller peer groups and more intimate bonds with others (Benenson, 2019; Vigil, 2007). In this sense, our participants have an additional layer of complication tied up in their concealment that male MAPs perhaps do not. Not only are they hiding their sexual attractions from their peers for risk of judgment or reprisal, but in doing so they are contravening the female norm of sharing personal details with trusted (typically female) peers (see Agrawal et al., 2002).

This concealment appears to have some negative impacts on psychosocial function within our sample. In describing herself as a “very very open person,” it is clear how Participant 2 contrasts this element of being secretive against the rest of her personality and life. The impact of her identity concealment is highlighted in the resultant effect of feeling that she is not being true to herself and “living a lie.” This is also echoed by Participant 1, who discusses feelings of “living deceitfully” as a result of her “deepest darkest secret.” This process of hiding important parts of one’s core identity, and the subsequent effects on the quality of social and personal relationships, echoes the narratives of male MAPs (Elliott & Lievesley, 2018; Freimond, 2013; Houtepen et al., 2016). That is, men experiencing minor attraction have also reported that withholding their sexual identities as MAPs left them feeling inauthentic with others. In not having authentic close relationships with others, those in our sample may be missing an important support mechanism via the achievement of the primary human good of relatedness or connectedness to other people (Harper et al., 2020; Ward & Stewart, 2003). That is, one prominent positive psychological model that has been applied to both forensic and clinical psychological contexts asserts that the achievement of a range of universally strived-for goals is important for the attainment of a “good life” (Barnao et al., 2016; Ward et al., 2007). In not having authentic relationships, our participants (and the MAP community more broadly) are not achieving a sense of social connectedness, which is a core part of living a healthy and happy life. Although Participant 1 acknowledges the negative feelings associated with hiding this part of herself, it is clear that she has considered the potential effects of disclosing their sexual attractions, such as losing her partner and being unhappy. These are common experiences for MAPs who do disclose to others (Freimond, 2013; Goode, 2010). For Participant 1, these costs are deemed to be too high, and so her sexual attraction toward children remains concealed from those closest to her.

For some participants, the hidden part of their identity, and the need to constantly keep this secret, impacted upon their health and wellbeing:

I was thinking well what is the point if I have to live a lie, if nobody knows the real me then what is the point living, there is just no point because all my relationships are just fake.

(Participant 1)

Lying to people I cared about made me feel like even more of a terrible person and contributed to my mental state with the depression and the anxiety.

(Participant 6)

Here, Participant 1 expresses the impact of keeping her sexual attractions a secret and the effect this had on her mental health at a time when she was considering suicide, emphasizing the emotional distress she felt. The extract projects a sense of feeling trapped, with the recognition that her life and relationships are false as a result of her secret, but unable to resolve or overcome this due to the potential consequences of disclosing her sexual attractions. From her perspective, an inauthentic life is not worth living, which signals that she has reached a point of exacerbation and could no longer continue. Similarly, Participant 6 recognizes the detrimental impact that lying to others had on her mental health, contributing to the negative sense of self that she already felt due to her attraction. These experiences are not uncommon within sexual minority groups, including among MAPs, with identity concealment being associated with poorer mental health outcomes (Gamarel et al., 2012; Lehavot & Simoni, 2011; Meyer, 2003; Pachankis, 2007; Pachankis et al., 2020). Recent work with MAP participants has also found significant levels of social isolation and loneliness that are correlated with psychological distress and suicidality (Elchuk et al., 2021). Relatedly, Lievesley et al. (2020) reported correlations between thought suppression and lower levels of wellbeing. Taken together, these data suggest that identity concealment, and the subsequent effects of perceived social isolation, can result in severe psychosocial outcomes for MAPs. These experiences are not rare among MAPs, with 46% of those with sexual attractions to children having previously considered suicide, and 67% of this subgroup stating that they were unable to speak to others about this (B4U-ACT, 2011b). As such, there appears to be a need to promote environments within which both male and female MAPs feel comfortable to disclose their sexual attractions and maintain authentic personal relationships with others.

## Alienation and Loneliness

Throughout the narratives, participants expressed feelings of being alienated and isolated because of their attractions.

Well it’s very isolating for one thing. I’m not really not very social but when I do, sex is a big topic and it’s something that people in their late 20’s early 30s talk about a lot and it’s very hard for me to relate to those conversations.

(Participant 5)

I can’t have the same discussions with them about like dating or about things because I don’t have any romantic interests in people my own age…I just can’t relate to like wanting to date people, having crushes on appropriately aged people so I can’t bond with my friends on those things.

(Participant 2)

The extracts above highlight how participants experienced feelings of isolation and being an outsider within their friendship group. For these participants, it appeared to hinder their relationships in a similar way to the experiences reported in the previous subordinate theme. However, here the process was not the conscious concealment of their identity, but more the recognition that they cannot “relate” or “bond” in the same way due to an inability to contribute to what they perceived to be normal discussions about sex and romantic relationships. It is worth noting that Participant 5 is exclusively attracted to children. This may play a role in the way she perceives herself within her social groups, as they are not able to draw on broader sexual attractions to adults as a method to mask her attractions to children. As such, she resorted to retreat from social interactions, which exacerbated feelings of difference.

It’s hard for me to not resent them at times because all my friends who are straight, gay or bi seem to have so much fun but I will never have that sort of experience of life which makes me sad at times.

(Participant 3)

It’s very frustrating like to know that I’m never going to be able to even so much as kiss on the lips somebody who I’m really attracted to and it’s kind of a frustrating, painful thing to think about and it’s something that people can’t really empathize with.

(Participant 5)

From the narratives it is clear how the comparison of themselves in relation to their peers leads to a range of difficult emotions, including resentment, frustration, and sadness linked to the stark awareness that they will never have the same experiences and enjoyment in life because of a sexual attraction pattern that they have not chosen for themselves, and that they are powerless to change. This supports previous theorizing that suggests that pedophilia (as a specific form of minor attraction) operates as a sexual orientation, being unchosen and remaining stable throughout life (Grundmann et al, 2016; Seto, 2012; though see Tozdan and Briken [2017, 2018, 2019]^2^). The added layer of frustration does not appear to link only to the sexual aspects on minor attraction, but also to the romantic aspects of being sexually attracted to children. Recent work by Martijn et al. (2020) found that self-reported prevalence of falling in love with children rose as a MAP’s attraction exclusivity increased (that is, exclusively minor-attracted individuals reported falling in love with children—but not with adults—at higher rates than those MAPs who were also sexually attracted to adults). Similarly, Dymond and Duff’s (2020) qualitative work highlights how the sexual aspects of being minor-attracted (i.e., the need for celibacy) are often easier to cope with than the experiences of romantic attraction to children. This is especially the case for Participant 5, who is exclusively minor-attracted. In this sense, it is often easier for people who are sexually attracted to children to suppress physical sexual desires than it is to ignore the emotional connections that they feel toward children. There are links here to positive psychological models of living a “good life,” where interpersonal connection and intimate relationships form a vital part of the human experience (Harper et al., 2020; Ward et al., 2007). The acknowledgment for some MAPs that sexual celibacy may also be combined with emotional disconnection through a lack of intimate partners leads to increased loneliness and exacerbated social isolation (Dymond & Duff, 2020; Elliott & Lievesley, 2018; Jahnke et al., 2015b; Jeske, 2016).

Participant 5 discusses an additional pain related to others’ lack of understanding or ability to relate to her difficulties, which for her are because she has not disclosed or shared her attraction with others. However, her comment also suggests a generalization of this point, in that others simply cannot empathize with such things. Similar research with male MAPs has found that non-disclosure of their sexual attractions led to participants feeling excluded in social settings, particularly in conversations about love interests or intimacy (Dymond & Duff, 2020; Freimond, 2013; Houtepen et al, 2016). These feelings further contribute to the suffering, feelings of alienation and isolation experienced by the participants.

For some, isolation is to some extent a choice:

It’s honestly very lonely to live with this attraction…Due to the anxiety that I feel towards my sexual interest, I prefer social isolation over potentially revealing my thoughts. I shut people out and refuse to talk to my therapist. So it does get lonely at times, and I imagine it will only get worse.

(Participant 3)

Participant 3 describes a strategy she uses to hide her attractions by consciously isolating herself, which both sacrifices social relationships and acts as a barrier to accessing appropriate therapy. Although she considers this a safer option than making people being aware of her sexual attraction, the result of this is loneliness and the associated risks of negative psychosocial effects (Elchuk et al., 2021). Goode (2010) stated that often MAPs are left to cope alone with their thoughts and feelings about their attractions, and that social support is a vital aspect of their maintenance of emotional health and stability. This isolation is exacerbated by an inherent mistrust of professionals—from whom support might otherwise be sought—regarding a fear or lack of clarity over reporting requirements (B4U-ACT, 2011a; Dymond & Duff, 2020; Grady et al., 2018; Levenson & Grady, 2019). As such, a common theme in the narratives of our participants was a general lack of social support or understanding from others that, when combined with a fear of discovery (Jahnke et al., 2015b), leaves them with no other option than to withdraw from relationships and try to cope alone.

### Disclosure Deterrent

Although participants discussed the negative effects of not disclosing their sexual attractions as depicted with the previous subthemes, the reasons for non-disclosure were clear throughout the narratives and were related to the perceived risks associated with doing so.

When it comes to discussing my sexual attractions, I can tell strangers anonymously but even that is difficult sometimes. I most certainly would never reveal it to my family or people I know because they would hate me. My older sister was sexually abused as a kid, so I don’t think my family would take too kindly to the idea of me being a pedophile. There’s that common misconception to get through first, the one where all pedophiles are child molesters.

(Participant 3)

If people knew who I really was then people would just run a mile and they would hate me. My partner, my family, my friends, they all would…everybody would desert me, and you know I probably end up homeless and a druggie or something. Those feelings were becoming more and more impossible to er ignore the feelings of what would happen if the people knew the real me.

(Participant 1)

The participant narratives here highlight the difficulty and fear associated with disclosure. For Participant 3, there is a recognition that even anonymous disclosure is not easy, though easier to overcome than the fear of disclosing to those who know her, with the participant confidently articulating how she would “never” consider this. This is likely due to the feared consequences of disclosure in that those she told would consequently “hate” her, which she attributes to a lack of understanding around minor attraction. With even anonymous disclosure being considered difficult, Participant 3’s narrative highlights the pervasive nature of societal stigma about sexual attractions to children (Jahnke et al., 2015a; Harper et al., 2018; 2021), in that MAPs are often reluctant to disclose these attractions even when they cannot be identified (Jahnke et al., 2015b). Similarly, a fear of hate and abandonment is what prevents Participant 1 from disclosing. As stated previously, there is a conflation of minor attraction (commonly referred to simply as “pedophilia”) and child sexual abuse. This is likely due to misreporting of child sexual abuse cases within the media, whereby such offenses (and those committing them) are ascribed the “pedophilia” and “pedophile” labels (Diefenbach, 1997; Harper & Hogue, 2017; Ischebeck et al., 2021; McCartan, 2010). Such reporting creates a heuristic in people’s minds, whereby the pedophile label intuitively evokes the thought of newsworthy predatory child sexual offenses (Harper & Hogue, 2015, 2017; McCartan, 2010). For Participant 3, fear related to this lack of understanding is further intensified by the sexual abuse experienced within her family, and a concern about what this might mean for how her family may begin to view her. Although this fear may be warranted in that members of the public do appear to ascribe traits related to abnormality and dangerousness to individuals identified as being minor-attracted (Jahnke, 2018), there is no evidence of *more* punitive attitudes toward this group among victims of child sexual abuse (or their families). Indeed, there is some evidence to the contrary, whereby in some cases abuse occurs within the home, which leads to the media stereotype of individuals who commit sexual offenses to be disrupted (Harper et al., 2017; King & Roberts, 2017; for a discussion of how the process of anti-stereotype humanization can decrease stigma toward MAPs, see Harper et al., 2018).

Other participants discussed the risks that prevent them from disclosing to those in a professional capacity:

I think if I went to a support group I just wouldn't feel safe being around other people who would look at me and know what I was…I don’t want to lose my family, I don’t want to lose my job, I don’t want to lose my home, I don’t want to lose my friends. I would lose everything. I know I would if that information went into the outside world.

(Participant 4)

Most health professionals have no experience with this and no decent understanding of mandatory reporting laws so you hear reports of people being investigated for just having these feelings and that’s not a risk that I will ever take.

(Participant 6)

In the narrative above, Participant 1 discusses why she would not feel safe accessing support, with her reasons being driven by a fear of the potential consequences of revealing her sexual attraction to others. This involves not only the loss of close relationships but also of life security, such as her home and employment. As such, she believes that she has “everything” to lose by disclosing. These concerns may have some accuracy when considering the experiences of many MAPs who have lost social connections after disclosing their attractions to them (Freimond, 2013; Goode, 2010), and echo the concerns of our participants reported in previous subordinate themes. However, a key difference here is that the participants are discussing the likelihood of seeking support in a professional sense. For Participant 6 this risk is the result of mistrust of health professionals due to a perceived lack of understanding around reporting laws. For both of these participants, the risks and potential consequences of accessing support greatly outweigh the benefits of seeking support. An anonymous survey by B4U-ACT (2011a) found that similar sentiments are shared by a majority of the MAP community, with 62% of respondents feeling that mental healthcare professionals would not keep their information confidential, potentially risking discovery and legal investigations (see also Dymond & Duff, 2020; Grady et al., 2018; Levenson & Grady, 2019). These concerns are not unfounded, with many health professionals being unclear about reporting requirements when working with MAPs with no offending histories (Beggs Christofferson, 2019). This may necessitate the development of best practice guidelines, adopted internationally, for ensuring that MAPs have access to non-judgmental, gender-responsive, and, importantly, confidential support when it is required (Levenson & Grady, 2019; Lievesley & Harper, 2021).

## Conclusion

In this paper we have presented what we believe to be the first in-depth analysis of the lived experiences of female MAPs. The themes emerging from participant narratives highlight a number of important consistencies between the experiences of our participants and the male MAPs recruited in previous work. For example, our participants reported how they feel that they cannot be open about their sexual attractions due to a fear of losing close friendships and family relationships (see also Dymond & Duff, 2020; Freimond, 2013; Goode, 2010; Houtepen et al., 2016). The narratives of the participants in this study further stress the importance of disclosure in the formation of a healthy identity within which they can integrate their sexual attractions and live a wholesome life with authentic personal relationships. However, they also identified how holding back this aspect of themselves can stop MAPs from accessing professional support for both emotional wellbeing-related issues, and (in some cases) in an effort to prevent them from acting upon their attractions (Grady et al., 2018; Houtepen et al., 2016).

Importantly, though, the first superordinate theme (“*A minority within a minority*”) highlights some unique challenges faced by female MAPs. However, there is a tacit assumption that this group may experience less stigma from society than males who are sexually attracted to children. However, our participants suggested that this lesser stigma only exists because of the lack of knowledge that female MAPs exist. This lack of knowledge extends to the MAP community too, meaning that the women in our sample felt like a minority within a minority, which further exacerbates their feelings of isolation. Even where our participants shared the experiences of their male counterparts, known sex differences in peer support styles (which relate to themes of alienation and identity concealment) may exacerbate these in women with sexual attractions to children. That is, although men might experience a sense of social alienation due to societal stigma (Elchuk et al., 2021; Jahnke et al., 2015a, 2015b), the male-typical social organization of larger and more emotionally distant peer groups (Vigil, 2007) might mean that these individuals are not missing the direct disclosure of issues related to their sexual attractions to the same degree as the women in our sample (for more a greater female tendency toward personal disclosures in peer relationships, see Agrawal et al., 2002).

Despite this feeling of intersectional alienation, it should be noted that participants felt that there were few gender-related differences between their experiences and those of male MAPs in the broadest sense. As emphasized by Participant 1 “there is not a lot of difference between men and women, we are all experiencing the same sort of distress and hatred from society.” Nonetheless, our participants acknowledged the importance of being able to find support by specifically engaging with other women who experience sexual attractions to children.

This study further highlights how current services for minor-attracted individuals are inadequate (B4U-ACT, 2011a; Cantor & McPhail, 2016; Seto, 2012; Levenson et al., 2019; Lievesley & Harper, 2021). Although wider society (and many aspects of the professional clinical community) might see the behavioral control of sexual attractions as a key concern (Jahnke, 2018; Stephens et al., 2021; Walker et al., 2021), this was barely mentioned by our participants. Instead, coping with the absence of romantic or intimate experiences, loneliness, poor self-concept, and stigma-related stress were more pressing worries (see also B4U-ACT, 2011a; Dymond & Duff, 2020; Elchuk et al., 2021; Jahnke et al., 2015b; Lievesley & Harper, 2021; Martijn et al., 2020), which again might indicate a difference based on gender. That is, the evolutionary psychology literature suggests that men are more inclined, on average, to pursue sexual variety and short-term mating opportunities than women, whereas women have a statistically greater desire for deeper romantic connections (e.g., Schmitt et al., 2012). Even within informal support spaces, female MAPs feel alienated from the majority of the rest of the MAP community because of their gender. It may be a positive step for those involved in such initiatives to develop women-only spaces for this population to have a specific place to share their experiences and offer peer support, free from the additional stigmatization that they might experience on mixed-gender forums.

One limitation of this present study was its reliance upon self-reported experiences. That is, participant narratives could have been influenced by a social desirability bias, such as a wish to avoid negative evaluations. This may influence the extent to which behavior-related difficulties were discussed in comparison with wellbeing or emotion-related concerns. It should also be noted that no participants were visible during the interview process as these were conducted via Skype audio (*n* = 5) and email (*n* = 1) which could present some limitations. For example, verification of their status as a female MAP was not possible. Although this lack of verification is an issue for any study wherein participants are not directly observable by the researchers as they provide data, it may be particularly important to note here in light of the small sample size and the importance placed in IPA on the analysis of participant narratives. Additionally, it could be argued that the loss of the non-verbal cues that are obtained in face-to-face data collection could impact the quality of the data collection and analysis. However, this is not considered essential for IPA where richness of the data relating to the participants experiences is a priority (Smith et al., 2009), with both methods employed here considered both suitable for achieving this (Curasi, 2001; Smith et al., 2009) and comparable to that of face-to-face interviews (Ratislavová & Ratislav, 2014).

This work sheds light on the need to consider gender as an important variable when seeking to understand MAP experiences. However, one limitation of our recruitment strategy was the self-selecting nature of the sampling, where we recruited female MAPs from online forums and social media. We did not set any inclusion or exclusion criteria about specific gender identities, nor did our interview schedule explore gender identity in a specific sense. There is a lack of current research into gender diversity within the MAP community. Future work might look to explore the intersections of gender diversity and sexual minority status within this population. This would also continue to move the area away from having a forensic focus and more toward exploring the experiences of living with sexual attractions to children in a more holistic sense (Elchuk et al., 2021; Jahnke et al., 2015b; Lievesley & Harper, 2021; Lievesley et al., 2020).

Furthermore, it is important to warn against the generalization of these findings due to the relatively small sample size. Nonetheless, the sample within this study is comparable to recent qualitative analyses of MAP experiences (e.g., Dymond & Duff, 2020), with the potential participant pool being further limited by the hidden nature of female MAPs within this population. However, it was never our aim to produce generalizable insights that apply to all female MAPs, nor is this ever an aim in qualitative research. Instead, we were interested in their lived experiences and have used these to identify themes that may be interesting to study further in subsequent confirmatory studies.

We have touched on some preliminary suggestions for future work, including the design of gender-responsive interventions and support services for women who are sexually attracted to children. This could include the formation of specific women-only boards on prominent support forums, where this group can discuss the specific challenges facing them, share advice, and communicate in a way that resembles the gendered norm of intimate peer discussions among women (Agrawal et al., 2002; Vigil, 2007). More fundamentally, though, there is a need to more comprehensively study women with such sexual attractions, exploring how known sex differences in social styles, relationship preferences, and sexuality interact with their sexual attractions to children. Methods of coping with sexual celibacy and a lack of intimate partners (or how adult–adult intimate relationships are managed within the context of their attractions to children) would be useful starting points for this research program. One potential stumbling block relates to access to a sample that is large enough to draw meaningful conclusions and generalizations. In the existing research on female MAPs, samples of just 42 (Tozdan et al., 2020) and 20 (Stephens & McPhail, 2021) have been available for analysis. In the current analysis we suggested that it may be that women do not recognize their sexual attractions to children or minimize incidental attractions due to an ability to maintain adult–adult sexual behaviors as a result of a higher degree of sexual fluidity than men (Diamond, 2016). As such, researchers might not focus on recruiting samples from established MAP-specific forums, but instead look at the prevalence of incidental, regular, and pervasive minor attraction among larger community samples of women (and indeed of men). Precedence for the viability of such large-scale analyses comes from Bártová et al.’s (2021) work into the prevalence of paraphilia in the general community. Reaching such a large sample to assess both paraphilic interests and behaviors should be considered a priority in this area of work. In doing so, more accurate prevalence estimates may be possible to calculate, and the extent to which minor attraction plays a role in women’s sexualities can be factored into analyses. In addition, although we recruited self-selecting women in this study, a recent informal investigation suggested that approximately 7% of the MAP population could have trans or non-binary gender identities (Herzog & Singal, 2021). Although this is not a peer-reviewed source and stems from a journalistic exercise, this figure would represent a significant minority of the MAP population and warrants further investigation into the additional intersections between sexual and gender identities among MAPs.

To conclude, this analysis of the lived experiences of female MAPs sheds light on the unique experiences of this population and provides a stepping-stone for further investigation. By adopting some of our recommendations in relation to responsive support design, the targeted recruitment of female MAPs in larger-scale quantitative work might explore both the convergence and divergence of needs and experiences among both male and female MAPs, including their experiences of stigmatization, their relative wellbeing, and the management of sexual attractions in everyday life. In doing so, we hope that this work inspires more targeted work to improve the design and implementation of effective services for assisting all MAPs in their pursuit of greater wellbeing and assists professionals in their goal of sexual abuse prevention.

## Footnotes

1 Due to the small number of female MAPs that exist within this community, we assured all participants that they would not be identifiable in research outputs. This included not revealing the exact forums from which they were recruited.

2 There is currently no consensus among the academic and clinical communities about whether minor attraction (or, more specifically, pedophilia) represents a form of sexual orientation. A detailed exploration of this debate is beyond the scope of this paper. However, we highlight this debate here as our participants’ experiences relate to one particular aspect of this debate (namely the immutability or changeability of sexual attractions to children).

## Declarations

### Data Availability

Data are not shared in this work in order to preserve the confidentiality of this population. Materials in the form of the interview schedule can be accessed from https://osf.io/xeshn/?view_only=013ebee24ff749b1955b0a264140b4cb.
